# Identification of biomarkers related to propionate metabolism in schizophrenia

**DOI:** 10.3389/fpsyt.2025.1504699

**Published:** 2025-04-02

**Authors:** Weiqi Xie, Zhihong Luo, Jiang Xiao, Xuehua Zhang, Chanjuan Zhang, Ping Yang, Liang Li

**Affiliations:** School of Clinical Medicine, Hunan Brain Hospital, Hunan University of Chinese Medicine, Changsha, Hunan, China

**Keywords:** schizophrenia, propionate metabolism, biomarkers, diagnosis, treatment

## Abstract

**Purpose:**

Schizophrenia (SCZ) is a severe mental disorder with complex etiology. Research shows propionate metabolism is crucial for neurological function and health. This suggests abnormalities in propionate metabolism may link to SCZ. Therefore, identifying biomarkers associated with propionate metabolism might be beneficial for the diagnosis and treatment of SCZ patients.

**Methods:**

SCZ datasets and propionate metabolism-related genes (PMRGs) from public databases were obtained. DE-PMRGs were identified through differential and correlation analysis of PMRGs. Machine learning was used to screen for key genes and validate expression levels, aiming to identify potential biomarkers. Gene Set Enrichment Analysis (GSEA) and immune infiltration analysis were performed on the biomarkers. An upstream regulatory network was constructed, and potential drugs targeting these biomarkers were explored. Finally, real-time fluorescence quantitative PCR (qPCR) was used to verify biomarker expression levels.

**Result:**

A total of 11 DE-PMRGs were identified, and machine learning technology was employed to further screen for 5 key genes. Among these, LY96 and TMEM123 emerged as potential biomarkers through expression verification. A diagnostic model was developed, achieving an area under the curve (AUC) greater than 0.7, which indicates strong diagnostic performance. Additionally, nomograms based on these biomarkers demonstrated promising predictive capabilities in assessing the risk of SCZ. To explore gene functions and regulatory mechanisms at a deeper level, a competitive endogenous RNA (ceRNA) regulatory network was constructed, including 2 biomarkers, 72 microRNAs, and 202 long non-coding RNAs. In addition, a regulatory network containing 2 biomarkers and 104 transcription factors (TFs) was also established to investigate the transcription factors interacting with the biomarkers. Potential biomarker-targeted drugs were identified by exploring the DrugBank database; notably, LY96 exhibited higher binding affinities for four drugs, with docking scores consistently below-5 kcal/mol. The qPCR results indicated that the expression levels of LY96 and TMEM123 in the whole blood of SCZ patients were significantly higher than those in the healthy control group, which was consistent with the results in the GSE38484 and GSE27383 datasets.

**Conclusion:**

This study identified disease diagnostic biomarkers associated with propionate metabolism in SCZ, specifically LY96 and TMEM123. These findings offer novel perspectives for the diagnosis and management of SCZ.

## 1 Introduction

Schizophrenia is a severe mental disorder with a complex etiology and global impact. It significantly affects patients’ social and occupational functioning (1, 2). The symptoms of SCZ encompass positive symptoms such as hallucinations and delusions, negative symptoms like apathy and anhedonia, and cognitive deficits, with cognitive impairment being a central feature of the illness (3–5). SCZ affects approximately 1.0% of the global population and substantially burdens individuals and their families (6). The exact cause of SCZ remains unclear, with evidence pointing towards a multifaceted interplay of genetic and environmental factors during critical stages of brain development (7). Current medical research focuses on identifying disease biomarkers to elucidate pathogenic mechanisms and guide diagnostic and treatment strategies. Recent studies have uncovered promising findings on objective biomarkers of SCZ, including genetic susceptibility genes, biochemical markers, brain imaging, electrophysiological features (such as abnormal eye movements or negative waves), retinal dysfunction, epigenetic changes, and gene-environment interactions (8). The role of metabolic processes in disease pathogenesis is of particular interest, focusing on how alterations in propionate metabolism impact overall health and disease progression.

Immune dysregulation plays a crucial role in the pathophysiology of schizophrenia. Research indicates impairments in both innate and adaptive immunity throughout the clinical course of the disorder, suggesting a pro-inflammatory state in certain patients (9). Elevated levels of inflammatory cytokines, such as IL-1β and TNF-α, have been detected in the serum of individuals with schizophrenia (10). These cytokines are associated with neuroinflammation, which can promote neurodegenerative processes and neuronal damage, potentially correlating with symptom severity and cognitive dysfunction (11–13). However, the precise mechanisms by which immune dysregulation contributes to schizophrenia remain under investigation (9, 14). Exploring inflammation-related biomarkers presents a particularly promising avenue of research. The presence of systemic inflammation in schizophrenia suggests that immune dysregulation may serve as a biological marker for the disease. Increased levels of C-reactive protein (CRP) and other inflammatory markers are associated with more severe psychosis and cognitive decline (9, 15). These biomarkers could assist in diagnosing and stratifying patients based on their inflammatory profiles, thereby facilitating more personalized treatment approaches. Furthermore, identifying specific immune cell types and their roles in the disease may reveal new therapeutic targets and enhance our understanding of the fundamental mechanisms underlying schizophrenia (16, 17).

Propionate, a short-chain fatty acid, is crucial in the human body (18). It is an intermediate product generated through intracellular glycolysis and fatty acid oxidation. Propionate can be converted into acetyl-CoA by pyruvate dehydrogenase and enter the tricarboxylic acid cycle, providing cells with the necessary energy. Its metabolism is intricately linked to key biological processes such as oxidative phosphorylation, cholesterol synthesis, lipid metabolism, and amino acid metabolism (19). Recent research has demonstrated that propionate plays a significant role in neurodegenerative diseases, with its neuroprotective effects mediated through signaling between neurons and the intestine. The aggregation of alpha-synuclein in neurons triggers the intestinal mitochondrial unfolded protein response (mitoUPR), which in turn reduces propionate production. Decreased levels of propionate lead to the downregulation of metabolism-related genes, resulting in intestinal energy deficits and exacerbating neurodegeneration via lactic acid and neuropeptide-mediated communication between the intestine and the brain (20). Furthermore, neurodegenerative and neuropsychiatric diseases, such as schizophrenia, share common signaling molecules and phenomena, including proinflammatory cytokines, γCaMKII, MAPK/ERK, chemokine receptors, blood-brain barrier (BBB) permeability, and the intestinal microbiota-brain axis (21). Consequently, it can be speculated that abnormal propionate metabolism may be linked to the development of schizophrenia.

Further research is required to explore the relationship between propionate metabolism and SCZ. However, current research in this area offers a fresh perspective on the pathophysiological mechanisms of SCZ. Potential biomarkers can be identified, thereby leading to new approaches for early diagnosis and treatment of SCZ by investigating the abnormal changes in propionate metabolism-related genes (PMRGs) in SCZ through bioinformatics.

## 2 Materials and methods

### 2.1 Source of data

The two data sets, GSE38484 and GSE27383, were obtained from the GEO database^1^ (22) for analysis. The GSE38484 data set, consisting of 106 SCZ patients and 96 normal control whole blood samples, was used as a training set. The GSE27383 data set, containing peripheral blood mononuclear cell (PBMC) samples from 43 SCZ patients and 29 normal HC, was used as a validation set. Furthermore, 16 genes associated with PMRGs were identified in the GeneCards database^2^ (23) through the keyword “Propionate metabolism”.

### 2.2 Differential expression analysis and screening of DE-PMRGs

Differential analysis was conducted using R software v4.2.2 and the limma software package (v1.38.0) (24). Genes meeting the criteria of |log2FC| > 0.5 and *P* < 0.05 were identified as differentially expressed genes (DEGs). The ggplot2 software package (v3.4.1) (25) was used to generate volcano plots and heat maps.

The GSE38484 dataset was used to analyze the expression differences of PMRGs between samples with schizophrenia (SCZ) and control samples. Boxplots were generated using the ggplot2 software package (v3.4.1). The difference in PMRG scores between SCZ samples and HC was computed using the GSVA software package (v1.42.0) (26). Significant differences in PMRG scores between SCZ and control samples were assessed using the Wilcoxon test. Spearman correlation analysis was conducted to determine the correlation between DEGs and PMRGs scores, with a correlation coefficient |r| > 0.5 and *P* < 0.05 as the criteria for identifying DE-PMRGs.

### 2.3 Functional enrichment analysis of DE-PMRGs

The distribution of DE-PMRGs on the chromosome was depicted using the Circos visualization tool. The relationship between these genes was then analyzed using Spearman correlation and visualized through a heat map. GO and KEGG pathway enrichment analysis was then conducted on DE-PMRGs using the clusterProfiler software package (v4.7.1.3) (27).

### 2.4 Screening biomarkers by machine learning

Lasso regression analysis was conducted on the candidate genes using the glmnet software package (v4.1.4) (28). In the Lasso model, the cross-validation method is employed to select the optimal regularization parameter, λ. Specifically, by minimizing the mean squared error (MSE), the optimal value of λ was determined in this study to be 0.0127193 (logλ =-1.8955). The Lasso coefficient spectrum and cross-validation error plots were generated to identify the key genes, which are those with regression coefficients not penalized to 0.

Box plots were generated to display the expression of key genes in SCZ samples and control samples from the training set GSE38484 and validation set GSE27383. Genes identified by their expression levels were noted as potential biomarkers.

### 2.5 Construction of diagnostic models and nomograms

Binomial logistic regression analysis was used to develop a diagnostic model using biomarkers from the training set GSE38484 and validation set GSE27383. The ROC package pROC was used to generate an ROC curve to evaluate the model’s efficacy in differentiating SCZ samples from control samples.

In the GSE38484 training set, biomarkers are used to predict disease status as the outcome event. The R package “rms” was used to develop a nomogram model based on these biomarkers, and a calibration curve was generated to assess the predictive performance of the nomogram model. The Hosmer-Lemeshow test (HL test) serves as an indicator of model fit by assessing the discrepancy between predicted and actual values. A p-value greater than 0.05 indicates that the model passes the HL test, suggesting no significant difference between the predicted and actual values. Conversely, a p-value less than 0.05 implies that the model fails the HL test, highlighting a significant discrepancy and indicating poor model fit. Furthermore, a Mean Absolute Error (MAE) of more than 0.05 indicates a small error between actual and predicted disease risks, reflecting the high precision of the nomogram model in predicting disease risk. Additionally, DCA curve analysis was conducted using the R package “rmda” to evaluate the clinical utility of the nomogram. To further assess the effectiveness of the nomogram, the ROC curve was plotted using the R package “PRROC” (29) and the model’s AUC was calculated to evaluate the model’s effectiveness.

### 2.6 GeneMANIA network construction and GSEA functional enrichment analysis

The GeneMANIA database^3^ (30) was used to construct and analyze the interaction network between biomarkers and their co-expressed genes. Correlations between biomarkers and all genes in the GSE38484 dataset were calculated, and the genes were ranked based on the correlation coefficient. GSEA enrichment analysis was subsequently conducted using the clusterProfiler software package (v4.7.1.3) with reference to the c2.cp.kegg.v2023.1.Hs.symbols.gmt gene set and c5.go.v2023.2 in the MSigDB database^4^ (31). The top three positive and negative pathways with enrichment scores were selected for visualization based on thresholds of |NES| > 1 and *P* < 0.05.

### 2.7 Immune cell infiltration analysis

The GSVA software package (v1.42.0) was used to assess immune cell infiltration in all samples from the training set GSE38484 using the ssGSEA algorithm. A box plot was generated to illustrate the distribution proportion of 28 immune cell types in the samples. The Wilcoxon test was used to compare differences in immune cell infiltration among the samples. A significance level of *P* < 0.05 was established for screening, and a box plot was created to visualize the immune scores between samples from individuals with SCZ and control samples. Spearman correlation analysis was used to investigate the relationship between biomarkers and differential immune infiltrating cells. A correlation with |r| > 0.4 and *P* < 0.05 was deemed statistically significant.

### 2.8 Construction of the upstream regulatory network

The TarBase database^5^ (32) was used to predict the miRNAs associated with the biomarkers, while the Starbase database^6^ (33) was used to predict the lncRNAs corresponding to the miRNAs. Subsequently, a ceRNA network consisting of lncRNA-miRNA-mRNA was established. Moreover, transcription factors (TFs) for the biomarkers were predicted using the ChEA3 database^7^ (34), where TFs with scores below 500 were selected, creating a TF-mRNA network. The visualization of both the ceRNA network and TF-mRNA network was done using Cytoscape software.

### 2.9 Construction and molecular docking of biomarker-drug interaction networks

The DrugBank database^8^ (35) was used to identify potential drugs targeting biomarkers, while Cytoscape software was used to visualize the mRNA-drug network. The 3D structure of the drug was obtained from the PubChem database^9^ (36), and the protein structure of the biomarker was retrieved from the PDB database^10^ (37). Molecular docking was then performed.

### 2.10 Clinical validation

#### 2.10.1 Subject recruitment

A total of fifteen schizophrenia patients hospitalized at Hunan Provincial Brain Hospital (Hunan Provincial Second People’s Hospital) between March 2024 and June 2024 were included in the experimental group. The inclusion criteria consisted of meeting the ICD-10 schizophrenia criteria; not having taken antipsychotic medications for one month prior to the onset of illness; being aged between 18 and 60 years; being capable of completing all scales; and providing written informed consent from a legal guardian. The exclusion criteria encompassed patients with mental retardation or organic brain diseases, as well as those with serious physical conditions such as heart, liver, or kidney diseases; patients exhibiting severely reduced social functioning or inability to cooperate due to psychiatric symptoms; and patients currently receiving psychological treatment. Additionally, fifteen healthy subjects who underwent physical examinations during the same period were selected as the control group. There were no significant differences in basic demographic information, such as gender (*P* = 0.635) and age (*P* = 0.642), between the two groups (**Table 1**). This research plan received approval from the Medical Committee of Hunan Provincial Brain Hospital (Approval Number: 2023K018). All participants provided informed consent at the beginning of the study.

**Table 1:** Comparison of the basic data of the two groups (Mean ± SD).

Parameter	HC(N=15)	Schizophrenia (N=15)	*P* value
Age (years)	34.20 ± 9.47	35.80 ± 8.78	0.635
Disease duration (years)	NA	7.73 ± 5.27	NA
Gender (Male/Female)	8/7	8/7	0.642
PANSS-total	NA	110.07 ± 3.65	NA
PANSS-positive	NA	30.20 ± 3.71	NA
PANSS- negative	NA	30.53 ± 2.97	NA
PANSS- general	NA	49.53 ± 2.03	NA

For analysis of difference between HC and schizophrenia group, independent samples t-tests and pearson’s chi-square test were used. Results were considered statistically significant at *P* < 0.05. SD, standard deviation; NA, not applicable.

#### 2.10.2 Quantitative reverse transcription polymerase chain reaction

Fasting venous blood samples were collected from subjects with SCZ and HC in the early morning. The samples were centrifuged at 4°C at 3000 r/min for 20 minutes, and the supernatant was retained. Total RNA was extracted from each sample using Trizol (Vazyme). Reverse transcription was conducted using HiScript II Q RT SuperMix for qPCR (+gDNA wiper) (Vazyme). Subsequently, quantitative PCR (qPCR) was performed using Taq Pro Universal SYBR qPCR Master Mix (Vazyme) on an ABI Q5 thermal cycler. The 2^−ΔΔCT^ method was employed to assess the relative expression levels between SCZ and HC for each selected central gene. The primer sequences utilized in this study are provided in **Table 2**. Details of the partial melting curves for the genes are included in the **Supplementary Figure 1**.

**Table 2:** Primer sequence list.

Gene	Primer	Sequence (5’-3’)	PCR Products
Homo GAPDH	Forward	TCCACTGGCGTCTTCACC	78bp
	Reverse	GGCAGAGATGATGACCCTTTT	
Homo LY96	Forward	CCCTGTATAGAATTGAAAGGATCC	124bp
	Reverse	TGCGCTTTGGAAGATTCATGGTG	
Homo TMEM123	Forward	GCTTCCACACAACTCCAGTGCT	121bp
	Reverse	ACTGGAGTCTGAGGCAACTGAAG	

### 2.11 Statistical analysis

R version 4.2.2 was utilized for all statistical tests. Figure panels were pieced together by EdrawMax. The significance of the correlation between the two groups was assessed through Spearman correlation analysis. Among patients and healthy volunteers, demographic and clinical behavioral data were compared using t-tests or chi-square tests, with a significance threshold set at *P* < 0.05. Data visualization was carried out using GraphPad Prism v.9.5.1 in conjunction with R version 4.2.2.

## 3 Result

### 3.1 Differentially expressed gene identification

**Figure 1:**
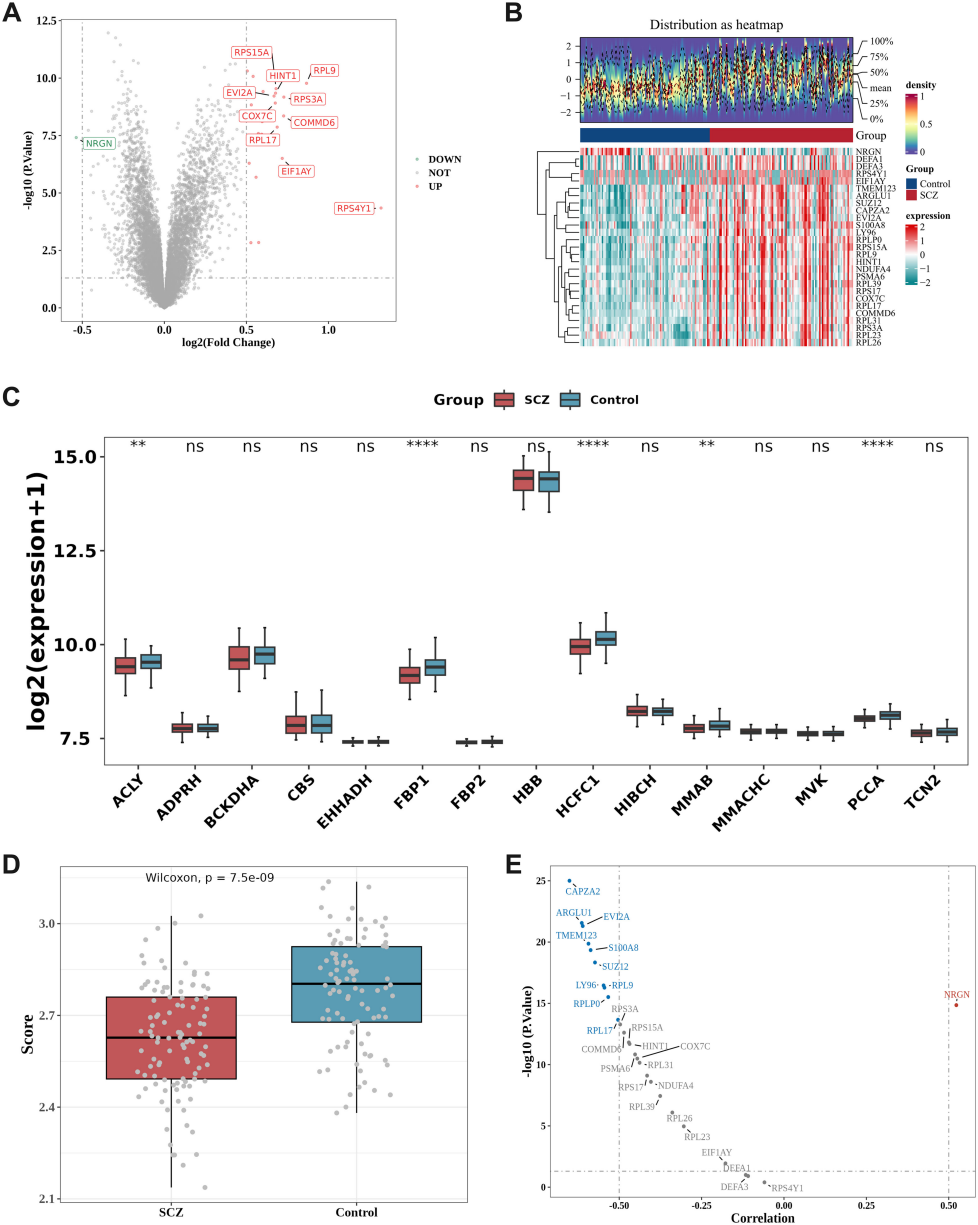
Differential expression analysis and correlation analysis to identify DE-PMRGs. **(A, B)** Differential expression analysis. Volcano plot **(A)** and Heatmap **(B)** of differentially expressed genes (DEGs) between SCZ and HC samples in the GSE38484 dataset. Heatmap: The upper section is a density heatmap of the expression levels of the top 10 up- and down-regulated genes, displaying lines for the five quantiles and the mean; the lower section is a heatmap of the expression of differential genes (with red representing high expression and green representing low expression); **(C)** Differences in propionate metabolism-related genes (PMRGs) in disease and HC. ns= no significant difference, **P < 0.01, ****P < 0.0001; **(D)** Differences in PMRGs scores in SCZ and HC; **(E)** Correlation volcano plot. ARGLU1, CAPZA2, EVI2A, LY96, RPL17, RPL9, RPLP0, S100A8, SUZ12, and TMEM123 exhibited negative correlations with PMRGs scores, whereas NRGN demonstrated a positive correlation with PMRGs scores.

The differential expression analysis results of the GSE38484 dataset revealed 27 differential genes between SCZ and normal HC, with 26 up-regulated genes and 1 down-regulated gene. The volcano plot (**Figure 1A**) displays the 10 most significant up-regulated and down-regulated genes, including RPS15A, RPL9, HINT1, EVI2A, RPS3A, COX7C, COMMD6, RPL17, EIF1AY, and RPS4Y1 for up-regulated genes, and NRGN for the down-regulated gene. Furthermore, the heatmap (**Figure 1B**) displays the expression levels of the 27 DEGs.

The boxplot (**Figure 1C**) illustrates the expression variances of PMRGs between samples from individuals with SCZ and HC samples. Among these, 5 PMRGs exhibited significantly higher levels in the control samples compared to the SCZ samples, specifically ACLY (*P* < 0.01), FBP1 (*P* < 0.0001), HCFC1 (*P* < 0.0001), MMAB (*P* < 0.01), and PCCA (*P* < 0.001).

The Wilcoxon test evaluated the GSVA scores of differentially expressed PMRGs between the disease group and the HC group within the GSE38484 dataset, revealing a significantly lower score in SCZ compared to the normal HC group (*P* < 0.05) (**Figure 1D**).

Spearman correlation analysis was conducted to assess the relationship between the DEGs and PMRG scores. Following the screening criteria of correlation coefficient |r| > 0.5, *P* < 0.05, a total of 11 DE-PMRGs were identified, including ARGLU1, CAPZA2, EVI2A, LY96, NRGN, RPL17, RPL9, RPLP0, S100A8, SUZ12, and TMEM123 (**Figure 1E**).

### 3.2 Functional enrichment analyses

**Figure 2:**
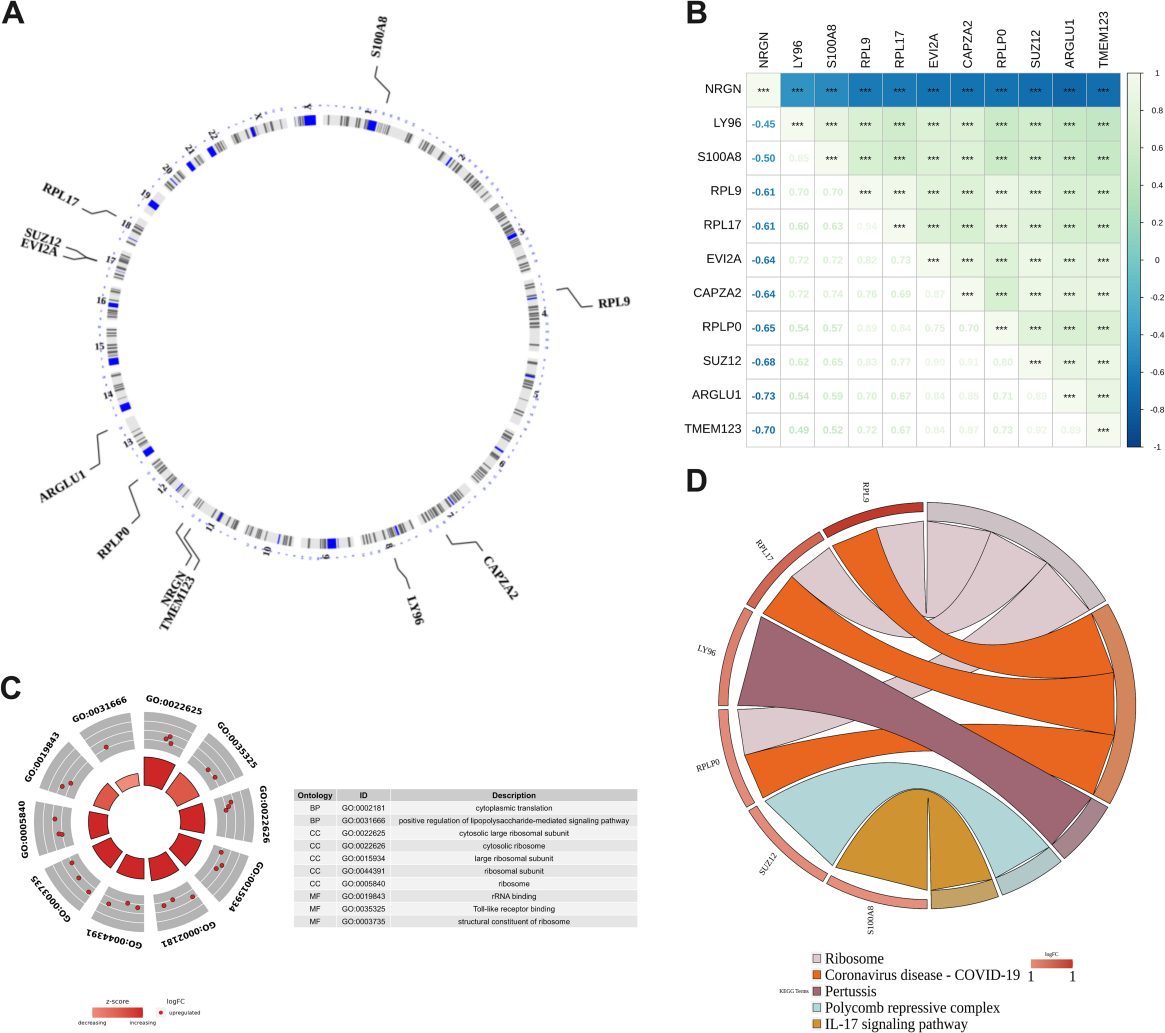
DE-PMRGs functional enrichment analysis. **(A)** Chromosomal location of DE-PMRGs; **(B)** DE-PMRGs correlation heat map, ***P < 0.001; **(C)** GO enrichment analysis of DE-PMRGs; **(D)** KEGG pathway analysis of DE-PMRGs.

The Circos heat map illustrates the distribution of 11 DE-PMRGs across different chromosomes. Specifically, S100A8 is situated on chromosome 1, RPL9 on chromosome 4, CAPZA2 on chromosome 7, LY96 on chromosome 8, TMEM123 and NRGN on chromosome 11, RPLP0 on chromosome 12, ARGLU1 on chromosome 13, EVI2A and SUZ12 on chromosome 17, and RPL17 on chromosome 18 (**Figure 2A**).

Spearman correlation analysis was conducted to assess the correlation between DE-PMRGs. The heat map (**Figure 2B**) revealed a significant correlation among all genes (*P* < 0.05). Notably, NRGN exhibited a significant negative correlation with other candidate genes (r <-0.4, *P* < 0.05), while the rest of DE-PMRGs showed a significant positive correlation (r > 0.4, *P* < 0.05).

GO and KEGG enrichment analyses were conducted on DE-PMRGs, resulting in a total of 109 outcomes from GO enrichment. The screening criteria used was *P* < 0.05, with 69 BP (biological processes), 21 CC (cell components), and 19 MF (molecular functions) being enriched. The enriched results were arranged in ascending order based on the *P* value, and the top 10 gene functions were presented (**Figure 2C**). Regarding biological processes, DE-PMRGs exhibited significant associations with cytoplasmic translation, positive regulation of lipopolysaccharide-mediated signaling pathways, response to glycoproteins, glial cell differentiation, and positive regulation of cell size. In terms of cell components, DE-PMRGs were notably linked to cytoplasmic large ribosomal subunits, cytoplasmic ribosomes, large ribosomal subunits, ribosomal subunits, and ribosomes. DE-PMRGs were found to bind to TOLL-like receptors, ribosomal structural components, ribosomal RNA, RAGE receptors, and lncRNAs for molecular functions. Furthermore, KEGG functional enrichment analysis identified 13 pathways, with the top 10 enriched pathways including ribosome, novel coronavirus, pertussis, polycomb protein inhibitory complex, IL-17 signaling pathway, NF-kappa B signaling pathway, TOLL-like receptor signaling pathway, toxoplasmosis, alcoholic liver disease, and motor protein pathway. The most significant enrichment results for pathways are illustrated in **Figure 2D**.

### 3.3 Screening for biomarkers

**Figure 3:**
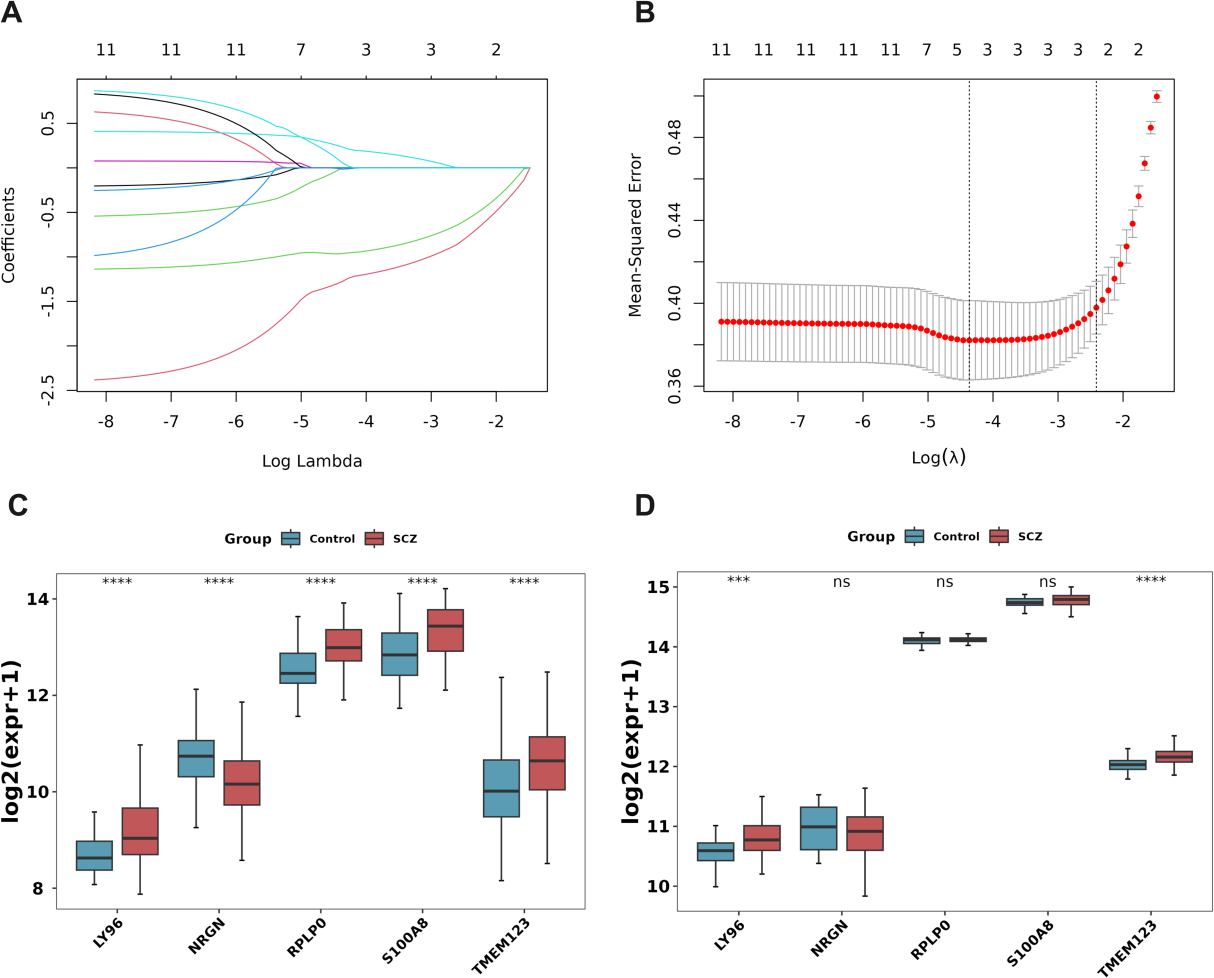
Machine learning and expression quantification for identification of biomarkers. **(A)** Lasso coefficient spectrum. The horizontal axis represents the logarithm of lambdas, and the vertical axis represents the variable coefficients, with each line representing a gene. As lambdas increase, the variable coefficients of the genes approach 0. When the optimal lambda is reached, variables with coefficients equal to 0 are eliminated; **(B)** Ten-fold cross-validation of adjusted parameters in LASSO analysis; **(C, D)** Key gene expression level verification. The training set GSE38484 **(C)**, the verification set GSE27383 **(D)**. ***P < 0.001, ****P < 0.0001. ns, no significant difference.

Lasso regression analysis was conducted on 11 DE-PMRGs (**Figures 3A, B**). 5 specific genes, namely LY96, NRGN, RPLP0, S100A8, and TMEM123, were identified as key genes as their regression coefficients were not penalized to 0.

The box plot shows the expression levels of key genes in SCZ samples and control samples in the training set GSE38484 (**Figure 3C**) and the validation set GSE27383 (**Figure 3D**). LY96 and TMEM123 were expressed similarly and significantly differently in these two data sets and were, therefore, identified as biomarkers.

### 3.4 Construction of diagnostic models and nomograms

**Figure 4:**
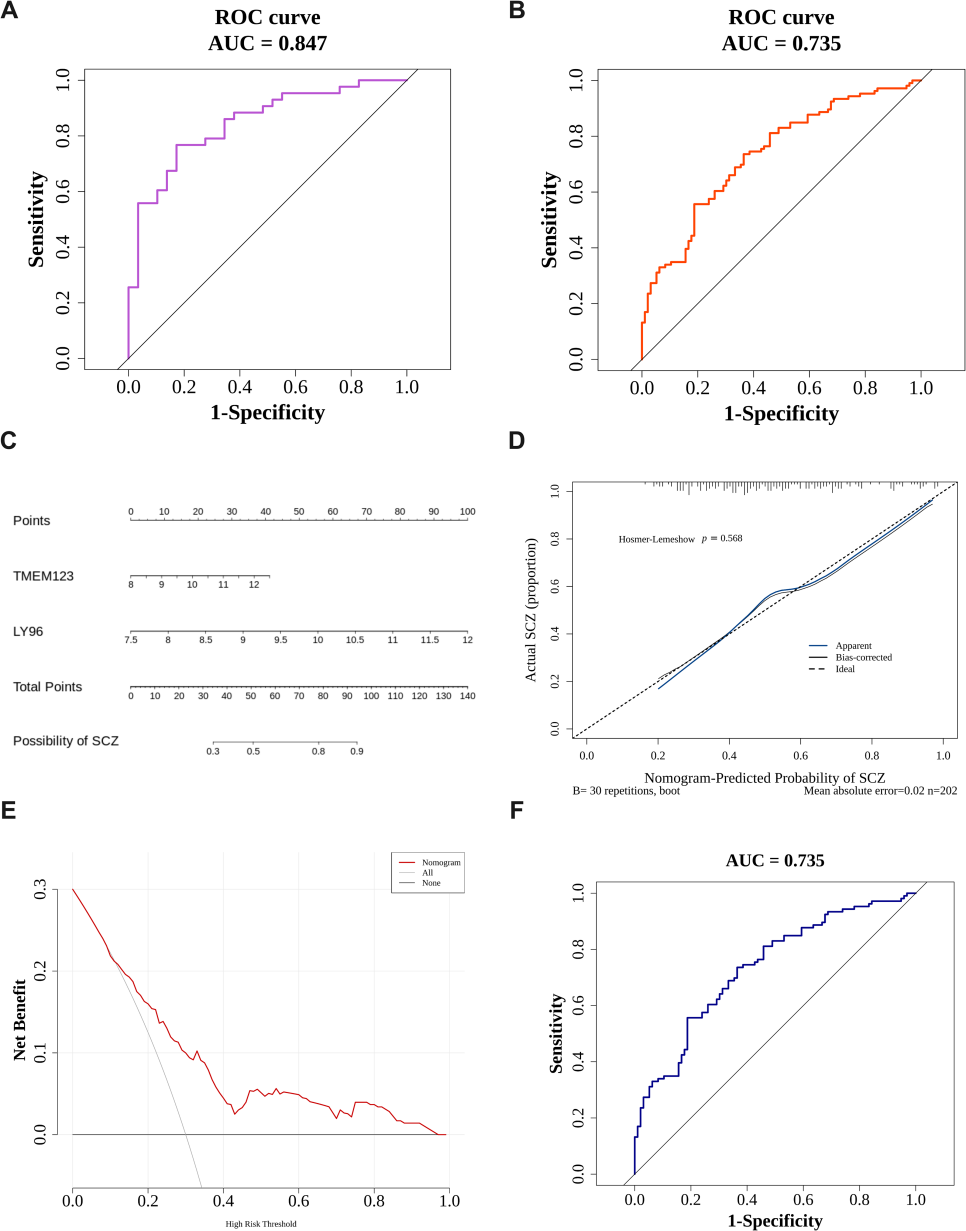
Constructing diagnostic models and nomograms. **(A, B)** Key gene logistic model ROC curve. The left picture is the training set GSE38484 **(A)**, and the right picture is the verification set GSE27383 **(B)**; **(C)** Creation of the nomogram for SCZ patients; **(D)** The calibration curve of the nomogram. **(E)** The DCA curve. The diagonal line (All) represents that all samples are intervened; the horizontal line (None) represents that none of the samples are intervened. **(F)** Nomogram model ROC curve.

A biomarker-based diagnostic model was constructed in the two data sets using binomial logistic regression analysis, and a ROC curve plot was drawn to evaluate and verify the effectiveness of the model in distinguishing SCZ and control samples. The results show that the AUC of the diagnostic model in the training set GSE38484 is 0.735 (**Figure 4A**), and the AUC in the validation set GSE27383 is 0.847 (**Figure 4B**). In both data sets, the AUC values of the logistic regression models constructed by LY96 and TMEM123 both exceeded 0.7, indicating a good diagnostic effect.

A nomogram (**Figure 4C**) was constructed to predict the probability of SCZ for samples in the training set GSE38484. The Hosmer-Lemeshow test was conducted to determine the dispersion between the predicted and true values. As shown in **Figure 4D**, the *P* value of the HL test is 0.568 (> 0.05), indicating no conspicuous difference between the predicted value and the true value. Moreover, the mean absolute error (MAE) is 0.02 (< 0.05), indicating that the error between the actual disease risk and the predicted disease risk is small and that the nomogram model has high accuracy in predicting the disease risk of the sample. In the DCA curve (**Figure 4E**), the nomogram model’s benefit rate, represented by the red line, significantly outperforms both the diagonal line (All) and the horizontal line (None). This indicates that, in comparison to the strategies of either treating all patients or none, the nomogram model offers additional benefits and demonstrates superior performance. Furthermore, in the ROC curve (**Figure 4F**), the model’s AUC exceeds 0.7, suggesting that the nomogram possesses a considerable level of predictive accuracy.

### 3.5 Biomarker analysis

**Figure 5:**
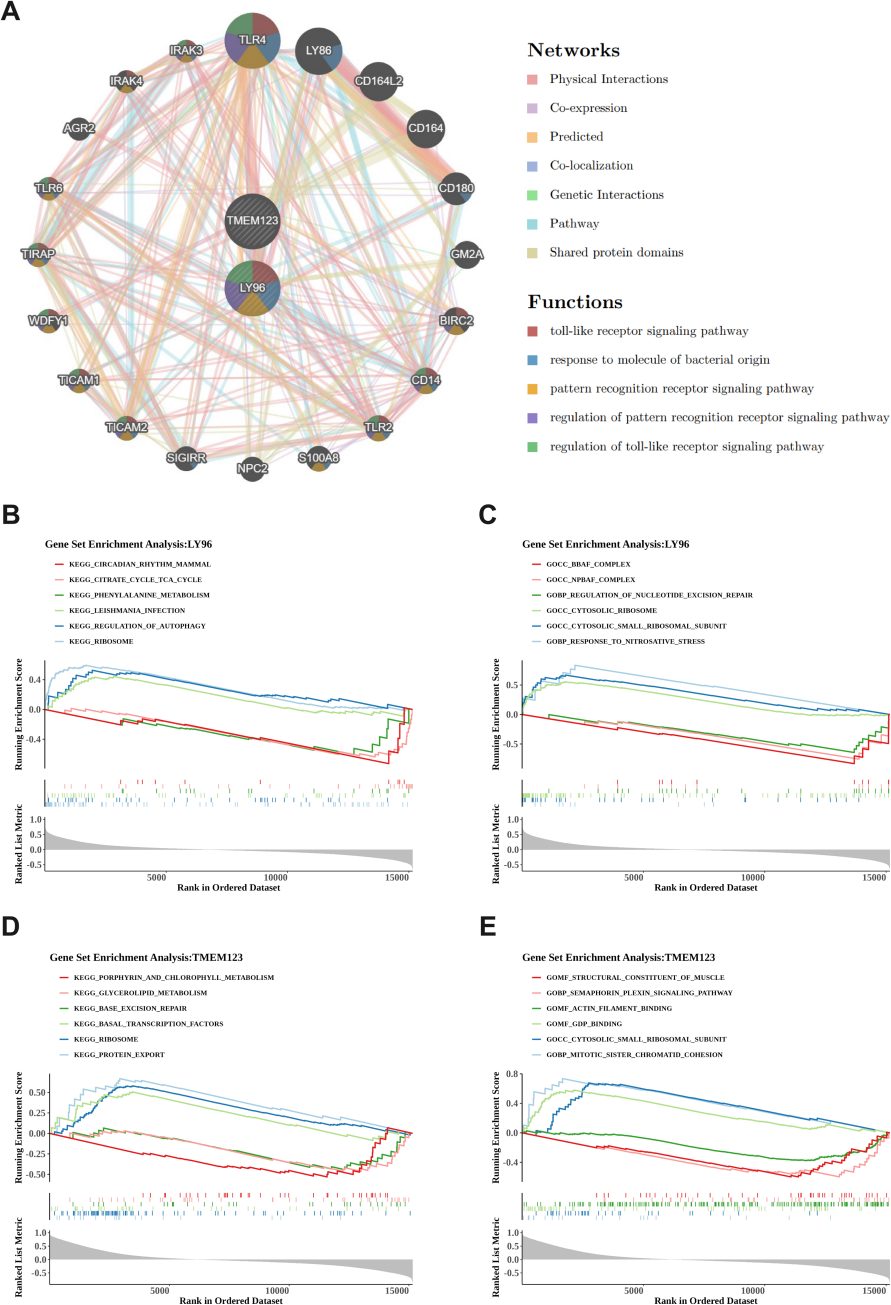
GeneMANIA network construction and GSEA enrichment analysis. **(A)**. GeneMANIA network of biomarkers. Identifying 20 interacting genes, with physical interactions constituting 77.64% and co-expression 8.01%, encompassing key functions such as the toll-like receptor signaling pathway and response to molecule of bacterial origin. **(B, C)** Functional analysis of LY96 gene. The left picture shows KEGG pathway analysis **(B)**, the right picture shows GO enrichment analysis **(C)**. LY96 is associated with 30 KEGG pathways including ribosomes and autophagy regulation, as well as 46 GO pathways; **(D, E)** Functional analysis of TMEM123 gene. The left picture shows KEGG pathway analysis **(D)**, the right picture shows GO enrichment analysis **(E)**. TMEM123 is associated with 18 KEGG pathways including protein export and ribosomes, as well as 34 GO pathways.

The GeneMANIA database was queried, and 20 genes interacting with the biomarkers LY96 and TMEM123 were obtained. **Figure 5A** shows the GeneMANIA network of biomarkers, including a total of 7 interactions between biomarkers and genes. Physical interactions account for the highest proportion (77.64%), followed by co-expression (8.01%). Moreover, among the biomarkers and their interacting genes, the top five related functions are the toll-like receptor signaling pathway, response to molecule of bacterial origin, pattern recognition receptor signaling pathway, regulation of pattern recognition receptor signaling pathway, and regulation of toll-like receptor signaling pathway.

The GSEA enrichment analysis results of LY96 and TMEM123 showed that KEGG of LY96 was enriched in 30 pathways and GO was enriched in 46 pathways; KEGG of TMEM123 was enriched in 18 pathways, and GO was enriched in 34 pathways. According to the threshold of |NES| > 1 and *P* < 0.05, the top three pathways with positive and negative enrichment scores were selected for picture drawing. KEGG enrichment analysis of LY96 found pathways such as ribosomes, autophagy regulation, Leishmania infection, phenylalanine metabolism, tricarboxylic acid cycle, and circadian rhythm disorders (**Figure 5B**). The GO enrichment analysis of LY96 includes pathways such as response to nitrosative stress, cytoplasmic small ribosome subunits, cytoplasmic ribosomes, regulation of nucleotide excision repair, npBAF complex, bBAF complex, etc (**Figure 5C**). KEGG enrichment analysis of TMEM123 revealed pathways such as protein export, ribosomes, basal transcription factors, base excision repair, glycerophospholipid metabolism, porphyrin, and chlorophyll metabolism (**Figure 5D**). GO enrichment analysis of TMEM123 involves pathways such as mitotic sister chromatid cohesion, cytoplasmic small ribosome subunits, GDP binding, actin filament binding, semaphorin plexin signaling pathway, and structural components of muscle (**Figure 5E**).

### 3.6 Immune infiltration analysis

**Figure 6:**
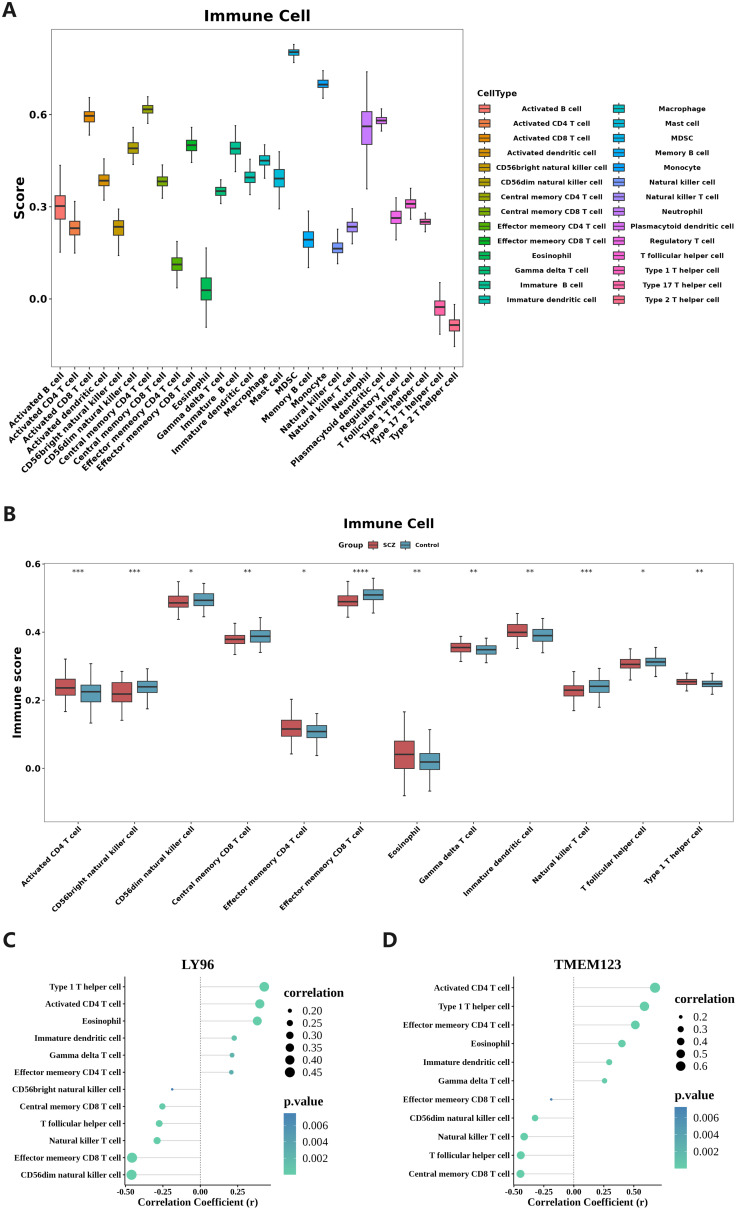
Immune infiltration analysis. **(A)** Relative abundance of 28 immune cells in the immune microenvironment of SCZ patients. The immune cell type with the highest relative abundance is MDSC, while the type with the lowest relative abundance is Th2 cells; **(B)** Twelve types of immune cells show significant variations between SCZ and HC samples. *P < 0.05, **P < 0.01, ***P < 0.001, ****P < 0.0001; **(C, D)** Correlation analysis of biomarkers and differential immune infiltrating cells. Both Type 1 T helper cells and Activated CD4 T cells, which are differentially infiltrating immune cells, exhibited significant positive correlations with the two biomarkers, LY96 and TMEM123.

**Figure 6A** illustrates the infiltration of immune cells in all samples from the training set GSE38484. The immune cell type with the highest relative abundance is myeloid-derived suppressor cells (MDSC), while the immune cell type with the lowest relative abundance is Th2 cells. **Figure 6B** presents the differences in immune cell infiltration between SCZ and control samples. Significant variations were observed in 12 types of immune infiltration cells between the disease group and the HC group, including activated CD4 T cells, CD56 bright natural killer cells, CD56 dark natural killer cells, central memory CD8 T cells, effector memory CD4 T cells, effector memory CD8 T cells, eosinophils, γδ T cells, immature dendritic cells, natural killer T cells, follicular helper T cells, and type 1 T helper cells. Then, Spearman correlation analysis was used to calculate the correlation between the biomarkers LY96, TMEM123, and differential immune infiltrating cells (correlation |r| > 0.4, *P* < 0.05 results were considered as significant correlation results).As depicted in **Figures 6C, D**, Type 1 T helper cell, Activated CD4 T cell, LY96, and TMEM123 all showed a significant positive correlation(r > 0.4, *P* < 0.05); LY96, CD56dim natural killer cell, and Effector memory CD8 T cell showed a significant positive correlation. Negative correlation(r <-0.4, *P* < 0.05), TMEM123 and Central memory CD8 T cell, T follicular helper cell, and Natural killer T cell showed significant negative correlation(r <-0.4, *P* < 0.05).

### 3.7 The competing endogenous RNA and TF-mRNA regulatory networks

**Figure 7:**
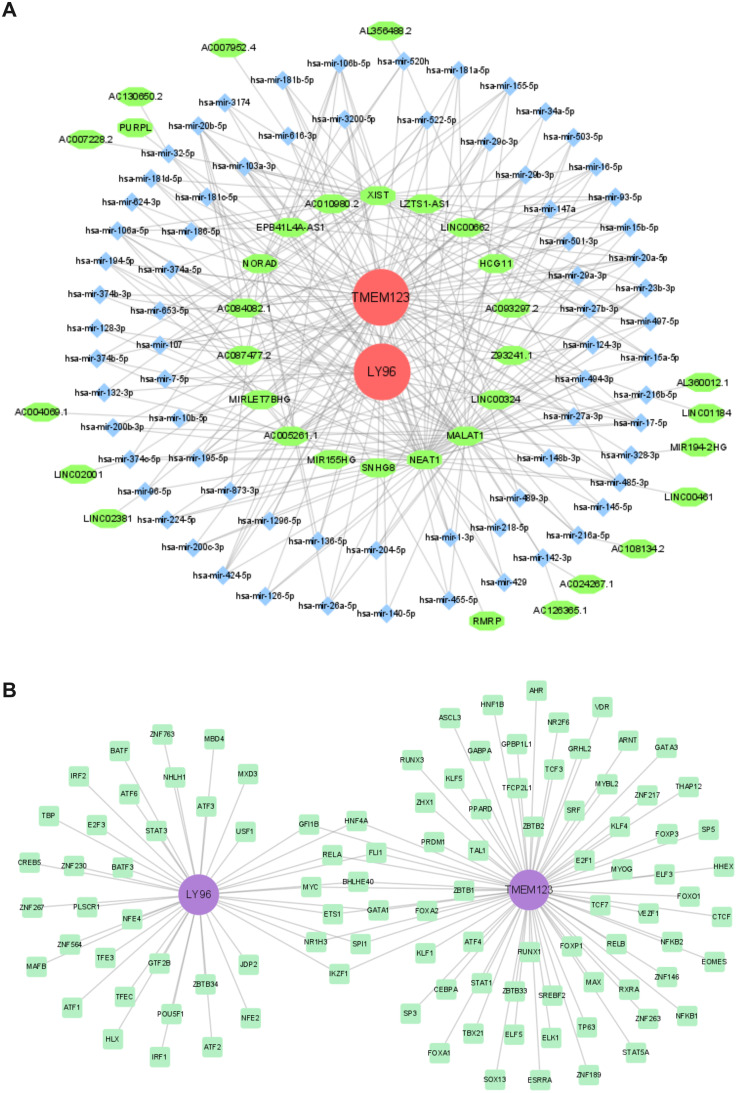
Regulatory network. **(A)**. CeRNA network; **(B)** TF-biomarker regulatory network.

The miRNAs of 72 biomarkers were predicted using TarBase. Among them, LY96 predicted 14 miRNAs, and TMEM123 predicted 67 miRNAs. The lncRNAs corresponding to miRNAs were predicted through Starbase. Among them, 72 miRNAs predicted a total of 202 relationships with corresponding lncRNAs and a total of 34 non-repeating lncRNAs. The construction of the lncRNA-miRNA-mRNA ceRNA network (**Figure 7A**) aids in understanding the gene expression regulatory mechanisms of biomarkers.

TFs for biomarkers were predicted using the ChEA3 database. LY96 predicted 42 TFs, and TMEM123 predicted 73 TFs, including 11 common TF transcription factors. A TF-biomarker regulatory network was constructed (**Figure 7B**).

### 3.8 Drug prediction-molecular docking

**Figure 8:**
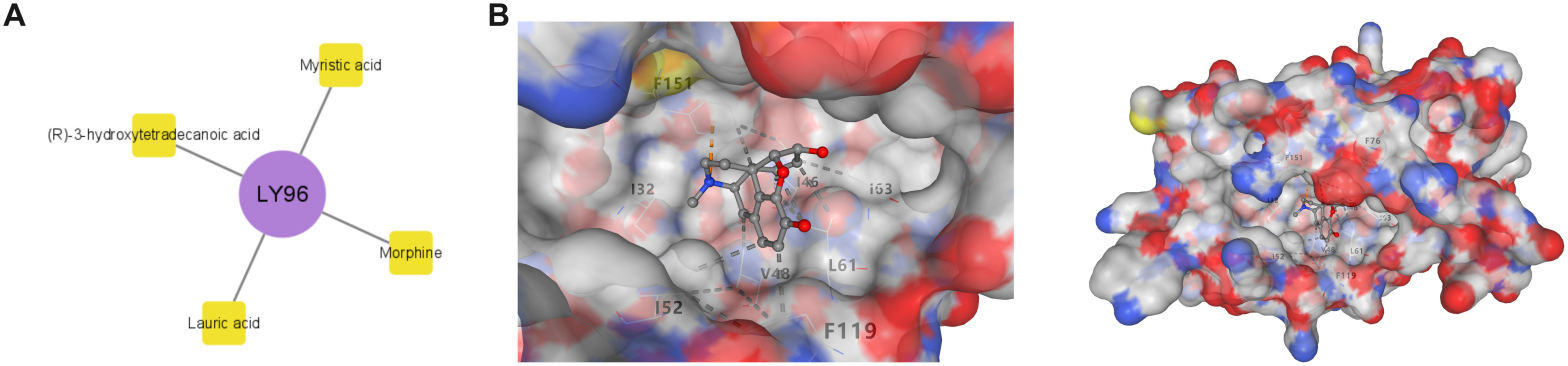
Drug prediction and molecular docking. **(A)** LY96 biomarker-drug network diagram; **(B)** Molecular docking of LY96 and Morphine.

Potential drugs targeting biomarkers LY96 and TMEM123 were searched for through the DrugBank database. LY96 predicted four drugs: Lauric acid, (R)-3-hydroxytetradecanoic acid, Morphine, and Myristic acid (**Figure 8A**). The 3D structures of these drugs and the protein structure of LY96 (PDB ID: 2E56) were obtained in PubChem and PDB databases, and molecular docking was performed on LY96 and the four drugs, respectively (**Figure 8B**, **Supplementary Figure 2**). A docking score below-5 kcal/mol indicates that the selected drug has a high binding affinity to the target. In **Table 3**, the docking scores of the four drugs and LY96 are all lower than-5 kcal/mol, indicating that small molecule compounds and key gene proteins have high binding affinity. Among them, the one with the lowest docking score was Morphine. **Figure 8B** shows the molecular docking of LY96 and Morphine.

**Table 3:** LY96-drug molecule docking scores.

Gene	Drug	Score
LY96	Lauric acid	-5.8
LY96	(R)-3-hydroxytetradecanoic acid	-6.0
LY96	Myristic acid	-6.0
LY96	Morphine	-7.6

### 3.9 Verification of biomarkers expression by qPCR

**Figure 9:**
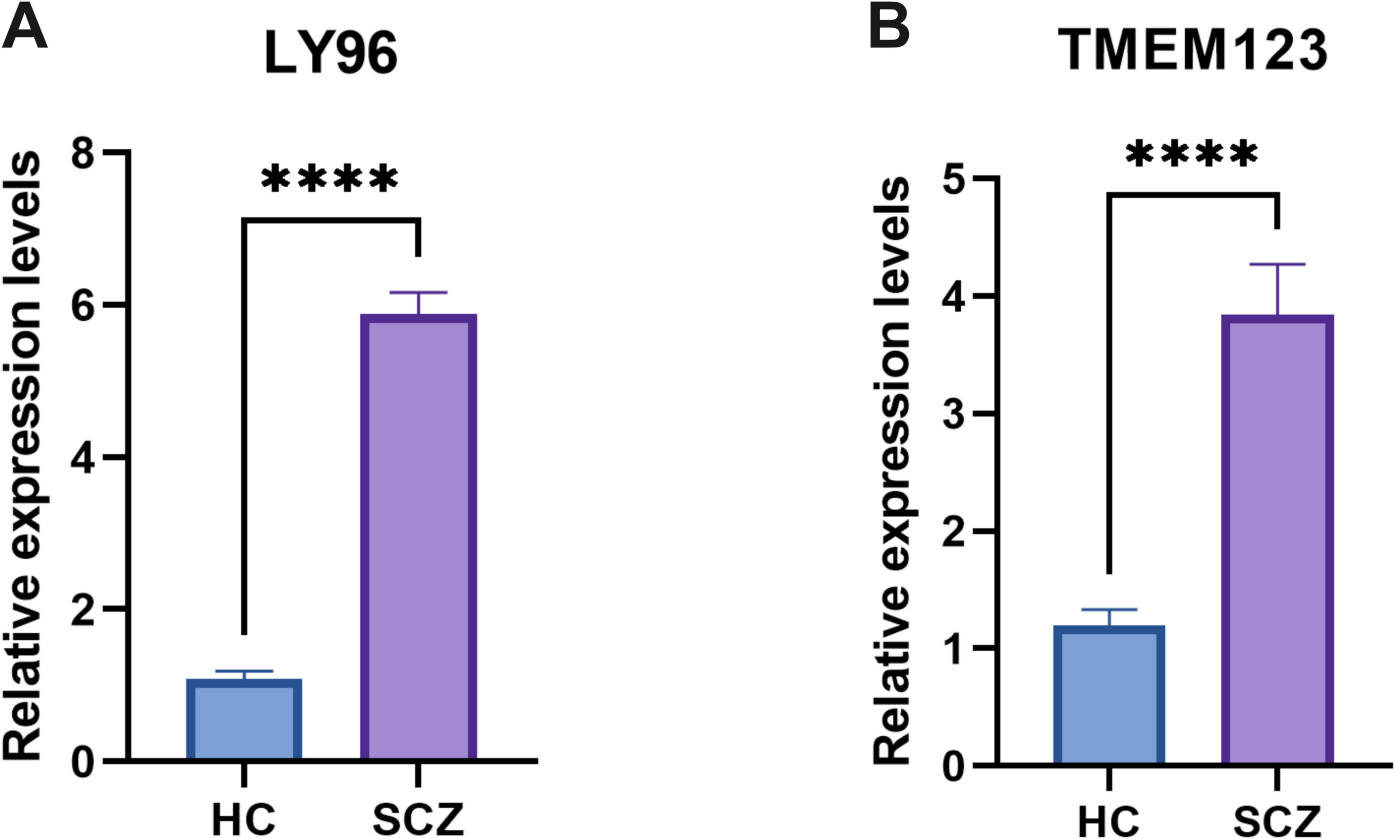
qPCR results showed that the expression levels of LY96 **(A)** and TMEM123 **(B)**. ****P < 0.0001.

The expression levels of the LY96 and TMEM123 genes in whole blood samples from patients with SCZ were assessed using quantitative polymerase chain reaction (qPCR). As anticipated, the results indicated that the expression levels of LY96 and TMEM123 in the whole blood of SCZ patients were significantly elevated compared to those in HC (**Figure 9**).

## 4 Discussion

Propionate is a short-chain fatty acid that is generated through intracellular glycolysis and fatty acid oxidation. It plays a crucial role in various biological processes, including the tricarboxylic acid cycle, oxidative phosphorylation, cholesterol synthesis, lipid metabolism, and amino acid metabolism (19), plays an important role in the human body (18). Previous studies have indicated that abnormal propionate metabolism is associated with neurodegenerative diseases such as Parkinson’s and Alzheimer’s. These conditions share common pathological features with schizophrenia, including pro-inflammatory cytokines, aberrant γ CaMKII, dysregulation of the MAPK/ERK pathway, increased blood-brain barrier (BBB) permeability, and dysregulation of the gut-microbiota-brain axis (21). This suggests a potential link between abnormal propionate metabolism and the development of schizophrenia. While existing studies have not delved into propionate metabolism in SCZ, this study has been the first to investigate abnormal propionate metabolism in SCZ patients. By analyzing 2 SCZ datasets and 16 PMRGs, DEGs were identified in patients and correlated with PMRGs to identify intersecting genes. Machine learning techniques were then used to identify key genes for expression verification, ultimately determining LY96 and TMEM123 as biomarkers associated with propionate metabolism in SCZ. Subsequent analyses included GSEA, immune infiltration analysis, construction of upstream regulatory networks, and molecular docking for drug prediction.

In this study, the validation dataset (GSE27383) comprised only male patients with SCZ and HCs, a characteristic that may limit the generalizability of the findings and subsequent analyses. This limitation restricts the study’s ability to fully represent the conditions experienced by female patients, particularly concerning gene expression, drug metabolism, and disease management. Females may exhibit distinct gene expression patterns and drug responses, as well as face gender-specific challenges such as pregnancy, childbirth, and menopause, which are not accounted for in male samples (38, 39). Future research should incorporate a more diverse patient population, including both males and females, to achieve a more comprehensive understanding of the differences in schizophrenia across genders.

LY96, also known as MD2, serves as a co-receptor for Toll-like receptor 4 (TLR4) by binding to its extracellular domain (40). The primary function of TLR4 is to detect lipopolysaccharide (LPS) derived from Gram-negative bacteria (41). LY96 is integral to this interaction (40). The TLR4-MD2 complex is situated on the cell membrane (42). Upon LPS recognition, this complex activates signaling pathways such as MyD88 and TRIF, which in turn initiate an inflammatory response (43) and stimulate the production of immune and inflammatory cytokines, as well as pro-inflammatory enzymes (41). Activation of the MyD88 pathway can lead to the activation of NF-κB and MAPKs (41), potentially exacerbating neurotoxic effects and promoting neurodegeneration (44). The TLR4-MD2 pathway may drive the progression of schizophrenia through multiple mechanisms. It promotes the activation of microglia (45) and the release of pro-inflammatory cytokines, such as IL-6 and TNF-α, resulting in chronic neuroinflammation and oxidative stress, which contribute to neuronal damage (46) and exacerbate positive symptoms, including delusions and hallucinations, as well as cognitive impairments. Additionally, the activation of this pathway upregulates dopamine synthase, resulting in excessive dopamine release within the mesolimbic system (47). Additionally, it inhibits glutamate uptake by astrocytes, which leads to synaptic glutamate accumulation and a reduction in NMDA receptor function (48, 49). These mechanisms are associated with the positive symptoms (50) and cognitive impairments (51) observed in schizophrenia. Furthermore, the activation of the TLR4 pathway in microglia contributes to excessive synaptic pruning, which may play a role in the onset of schizophrenia (52). Regarding treatment, TLR4 inhibitors such as TAK-242 and baicalin have demonstrated potential in mitigating neuroinflammation and depressive-like behaviors (53). Clozapine and minocycline exhibit anti-inflammatory effects (54, 55), and combining inflammatory marker detection can optimize therapeutic efficacy. However, it is necessary to balance the risks of immunosuppression and overcome the delivery limitations of the blood-brain barrier. Our study reveals that LY96 expression is significantly elevated in schizophrenia patients compared to control samples, thereby supporting the involvement of LY96 in LPS-induced TLR4 signaling pathway activation and the pathogenesis of schizophrenia.

TMEM123, also known as PORIMIN or KCT3, is a gene located in the q22.2 region of chromosome 11. TMEM123 encodes a transmembrane protein that plays a key role in cell communication, signal transduction, and substance transport. Research indicates that TMEM123 can induce tumor-like cell death in Jurkat cells (56). Our research found that the expression of TMEM123 in patients with schizophrenia is significantly higher than in the HC group, but there is currently insufficient evidence to support its reliability and specificity as a biomarker.

KEGG enrichment analysis revealed that LY96 is associated with pathways including ribosomes, regulation of autophagy, phenylalanine metabolism, tricarboxylic acid cycle, and circadian rhythm disorders. Similarly, TMEM123 was found to be linked to pathways such as ribosomes, basal resection repair, and glycerophospholipid metabolism. Patients with schizophrenia in the European population exhibit elevated levels of ribosomal DNA (rDNA) in their blood (57, 58). The transcriptional activity of rDNA may be a significant factor in neuronal plasticity, with dysfunctional neuroplasticity being considered a crucial pathophysiological mechanism of schizophrenia and being linked to genetic factors (59). Autophagy plays an important role in SCZ, involving neuronal homeostasis, pathophysiology, and symptom regulation (60), in which the regulation of autophagy-related genes (ARGs) plays a key role. Genetic variations in ARGs, including polymorphisms or mutations, may disrupt the normal process of autophagy and increase susceptibility to SCZ (61). Dopamine is the main neurotransmitter related to the neurobiology of SCZ. Phenylalanine hydroxylase (PAH) catalyzes the conversion of phenylalanine (Phe) to tyrosine (Tyr) in the brain. These two amino acids are a precursor for the synthesis of dopamine (62). Studies have shown that SCZ patients have PAH activity impairment (63), causing increased Phe levels and decreased Tyr levels in plasma (64–67), affecting the production of dopamine and leading to nervous system disorders. The intermediate products of the tricarboxylic acid (TCA) cycle may indirectly influence the onset and progression of schizophrenia by modulating neurotransmitter synthesis. Alpha-ketoglutarate, a pivotal molecule linking the TCA cycle to the glutamate-GABA-glutamine cycle, has been shown that its reduced levels can directly inhibit GABA synthesis (68, 69). GABA, the principal inhibitory neurotransmitter in the brain, plays a crucial role in the pathophysiology of schizophrenia (70). SCZ is closely related to circadian rhythm disorders, including abnormal staging, instability, and discontinuity of sleep-activity rhythms (71–74). The two often occur simultaneously and may involve the same brain mechanisms (75). The XRCC1 protein plays a key role in the base excision repair process and is closely related to the susceptibility to SCZ (76). Excessive oxidative stress triggers phospholipid remodeling, disrupts membrane lipid homeostasis, causes membrane dysfunction, and leads to SCZ progression (77). Glycerophospholipids are the most common component of biological membranes, and their metabolism is reduced in SCZ patients (78). This abnormality plays a key role in the disease progression.

GO enrichment analysis revealed that LY96 is associated with the response to nitrosative stress, regulation of nucleotide excision repair, npBAF complex, and bBAF complex, among others. Additionally, TMEM123 is linked to the semaphorin plexin signaling pathway. Nitrosative stress is mediated by nitric oxide (NO) released from NO synthase (NOS). It has emerged as a crucial signaling molecule in schizophrenia (79). Based on this, the increase in nitrosative stress or reactive nitrogen associated with abnormal NO levels is related to neuronal damage in SCZ (80, 81), and is considered an emerging pathological process in the disease (82). The XPC gene, involved in the early stages of nucleotide excision repair (NER) (83), may be impacted by XPC polymorphisms, leading to compromised DNA repair mechanisms and heightened SCZ susceptibility (84). The BAF complex plays a critical role in neural development by influencing neural fate and function. Mutations in BAF complex subunits have been associated with neurodevelopmental disorders, including SCZ (85). Semaphorins act as ligands for plexins and are involved in the plexin signaling system. Research indicates that variations in the SEMA3D gene may contribute to SCZ pathogenesis by influencing neural network development (86). This suggests that abnormalities in semaphorin plexin signaling pathways could increase the risk of neurodevelopmental disorders in SCZ patients.

In immune infiltration analysis, significant differences were noted in 12 types of immune cells between the disease group and the control group. The correlation between the two biomarkers and these immune cells was then analyzed. The results showed that type 1 T helper cells, activated CD4 T cells, significantly correlated with LY96 and TMEM123, indicating that these two differential immune cells may be involved in pathogenesis of SCZ. LY96 is a key accessory molecule of the TLR4 signaling pathway. Activation of the TLR4 signaling pathway can promote the secretion of cytokines such as interferon-gamma (IFN-γ), thereby enhancing the immune function of Th1 cells. At the same time, activated CD4 T cells can further differentiate and proliferate, enhancing the body’s cellular immune response (87). In addition, the TLR4 signaling pathway promotes the secretion of IL-12 (88) and induces the differentiation of naive CD4+ T cells towards the Th1 lineage through the STAT4 signaling pathway (89), which may lead to Th1/Th2 imbalance, causing abnormal immune responses and subsequently affecting the function of the nervous system, related to the occurrence and development of schizophrenia (90). On the other hand, TMEM123 is involved in cell death and immune regulation and may play a role in T cell activation and apoptosis. It regulates the survival and function of T cells by controlling cell death, thereby modulating the activity of Th1 and activated CD4+ T cells, indirectly participating in the occurrence and development of schizophrenia.

The ceRNA network links the functions of protein-coding mRNAs with those of non-coding RNAs, such as microRNAs, long non-coding RNAs, pseudogene RNAs, and circular RNAs. lncRNAs can competitively bind to shared miRNAs, and their expression levels are positively correlated. The upregulation of an lncRNA can sequester more shared miRNAs (91). In our study, a ceRNA network was constructed through LY96 and TMEM123, indicating that this regulatory network plays a role in schizophrenia, which provides direction and evidence for further elucidating the mechanisms of these biomarkers in SCZ.

## 5 Conclusion

In summary, this study established a connection between propionate metabolism-related genes SCZ through differential analysis, GSVA, GSEA, and other methodologies. The biomarkers LY96 and TMEM123 were identified and demonstrated a good ability to distinguish patients with SCZ from HC. This work lays the groundwork for further exploration of the role of propionate metabolism-related genes in SCZ.

## Data Availability

The original contributions presented in the study are included in the article/**Supplementary Material**. Further inquiries can be directed to the corresponding authors.

## Footnotes

1
https://www.ncbi.nlm.nih.gov/geo/

2
https://www.genecards.org

3
http://genemania.org/

4
https://www.gsea-msigdb.org/gsea/msigdb

5
http://www.tarbase.com/

6
http://starbase.sysu.edu.cn/

7
https://maayanlab.cloud/chea3/

8
https://go.drugbank.com/

9
https://pubchem.ncbi.nlm.nih.gov/

10
https://www.rcsb.org/

## Supplementary material

Supplementary Figure 1 Amplification curve and melting curve of Q-PCR. **(A)**. Amplification plot of Homo GAPDH; **(B)**. Melt curve plot of Homo GAPDH. **(C)**. Amplification plot of Homo LY96; **(D)**. Melt curve plot-Homo LY96; **(E)**. Amplification plot of Homo TMEM123; **(F)**. Melt curve plot of Homo TMEM123.

Supplementary Figure 2 Molecular Docking. **(A)**. Molecular Docking of LY96 and (R)-3-hydroxytetradecanoic acid; **(B)**. Molecular Docking of LY96 and Lauric acid; **(C)**. Molecular Docking of LY96 and Myristic acid.
