# Supplementary figures and images for: Identification of biomarkers related to propionate metabolism in schizophrenia
Source: Front Psychiatry. 2025 Apr 2;16:1504699. doi: 10.3389/fpsyt.2025.1504699 (PMC12000038; doi:10.3389/fpsyt.2025.1504699)

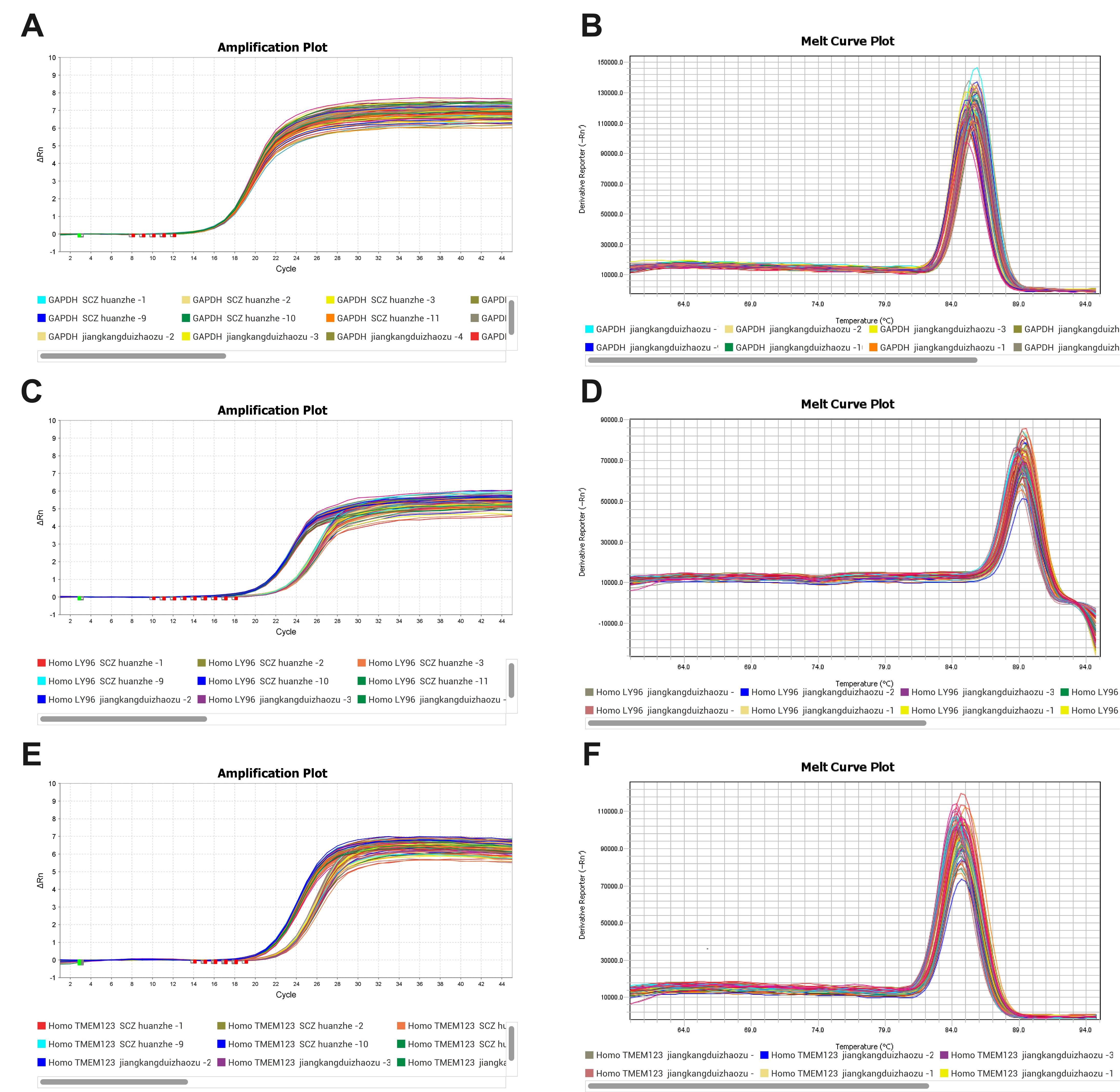

Supplement: Supplementary Figure 1 — Amplification curve and melting curve of Q-PCR. (A). Amplification plot of Homo GAPDH; (B). Melt curve plot of Homo GAPDH. (C). Amplification plot of Homo LY96; (D). Melt curve plot-Homo LY96; (E). Amplification plot of Homo TMEM123; (F). Melt curve plot of Homo TMEM123. [file Image1.tiff]

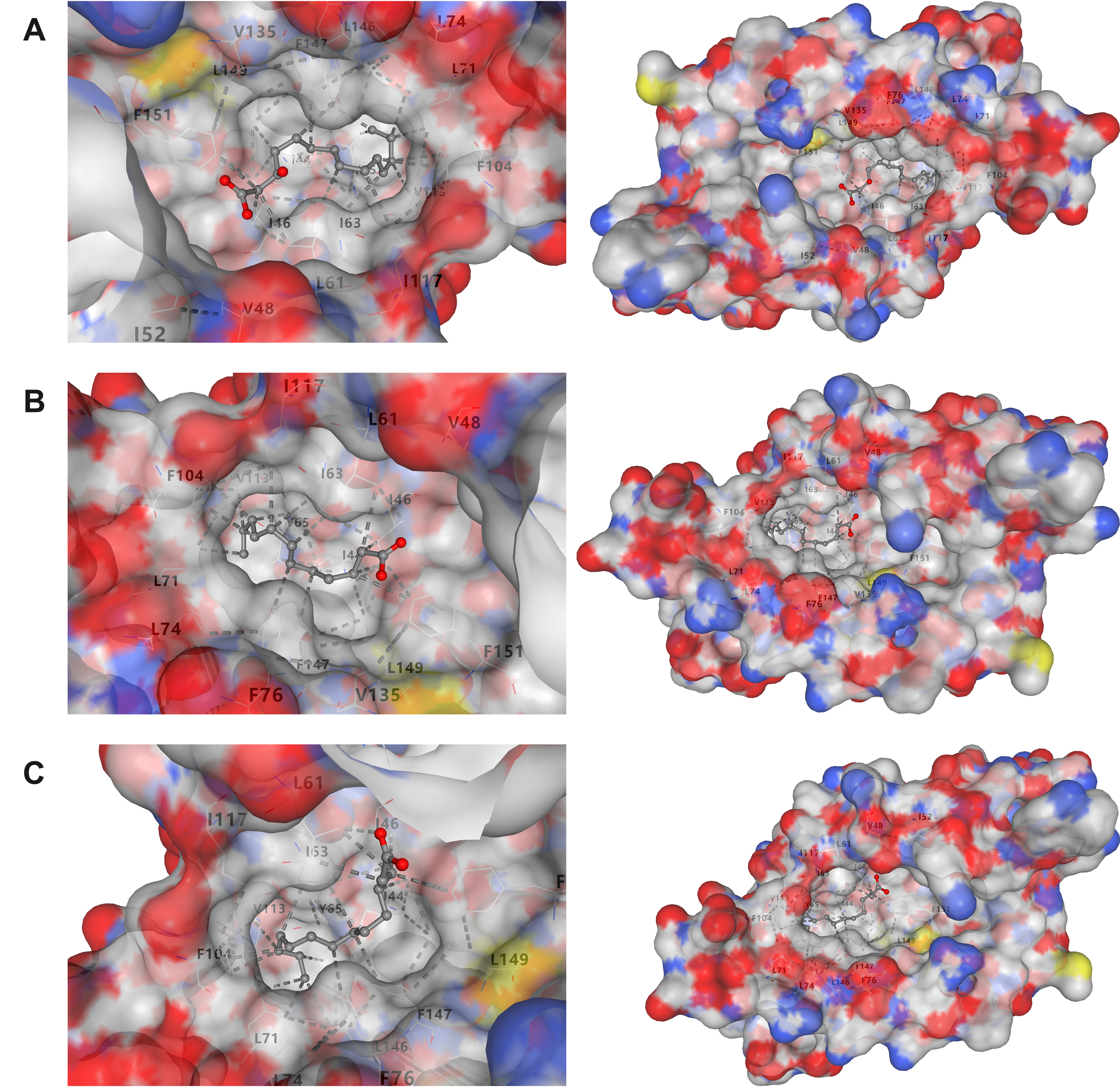

Supplement: Supplementary Figure 2 — Molecular Docking. (A). Molecular Docking of LY96 and (R)-3-hydroxytetradecanoic acid; (B). Molecular Docking of LY96 and Lauric acid; (C). Molecular Docking of LY96 and Myristic acid. [file Image2.tiff]
